# Editorial: Internet Addiction & Gaming Disorders in Children and Adolescents

**DOI:** 10.3389/fpsyt.2022.870177

**Published:** 2022-03-15

**Authors:** Kirthana Vasudevan, Daniel Shuen Sheng Fung

**Affiliations:** Department of Child and Adolescent Psychiatry, Institute of Mental Health, Singapore, Singapore

**Keywords:** internet, addiction, social media, child, adolescent, child psychiatry

## Introduction

Communications has always been the main driver of the globalized world. The Internet is a means which allows global sharing of knowledge, news, and entertainment. It has enabled intersectoral exchange in education, healthcare, and business. The recent COVID-19 pandemic has also emphasized the importance of the virtual world in telemedicine, home based learning, and telecommuting (1, 2). The digital generation has raised concerns about screen times (3), Internet addiction [Yu and Shek; Siste et al.; Fung et al.; Nik Jaafar et al.; (4)] as well as potential risks in social media [Yu and Shek; (5)]. Computer addiction was a concern as early as the 1980s (6).

## Evaluation of Internet Addiction/Gaming Disorders

The International Classification of Diseases (ICD-11) has recognized gaming disorder to be one that is characterized by (a) impaired control over gaming, (b) increasing priority given to gaming over other activities to the extent that gaming takes precedence over other interests and activities, and (c) continuation of gaming despite experiencing negative consequences. It is also defined to be severe enough to cause significant functional impairment in various aspects of one's life (7). However, The American Psychiatric Association has placed “Internet Gaming Disorder” under Section III of Diagnostic and Statistical Manual of Mental Disorders (DSM-5), as a condition that warrants more clinical research (8). Related maladaptive behavior such as excessive use of Internet or social media, and their associated impact on mental health issues need further exploration.

A holistic approach to clinical evaluation of disorders associated with Internet or gaming addiction would involve (a) clear definition of problematic behaviors and disorders, (b) use of accurate diagnostic tools (c) identification of risk factors, and (d) identification of protective factors.

## The Need for Clear Definition

The prevalence of maladaptive behavior patterns associated with Internet use has garnered global interest. A study in Hong Kong showed that 11.4% of adolescents (*n* = 1,896) had social networking addiction (Yu and Shek.) A study in Indonesia showed that 19.3% of adolescents (*n* = 2,932) were determined to have Internet addiction (Siste et al.). A South Korean study involving 2,984 adolescents explored defining various forms of Internet users, including gamers. Overall, 7 different profiles were described. These profiles showed variation in gender, problematic gaming behavior, as well as neuroticism (Kim et al.). These studies suggested that addictions are associated with various digital forms and need to be differentially identified in clinical assessment.

## The use of Accurate Diagnostic Tools

Since the definition of Internet addiction continues to evolve, it becomes challenging to adopt universal diagnostic tools. A meta-analysis that studied the reliability and validity of 5 Gaming Disorder Scales (GAS-7, AICA, IGDT-10, Lemmens IGD-9, and IGDS9-SF) showed that they had good internal consistency and test-retest reliability. However, few studies had checked for test-retest reliability (9). A China-based study evaluated the reliability and internal consistency of the “Chinese Internet Gaming Disorder Checklist (C-IGDC)” in determining the presence of Internet gaming disorder (Chen et al.). We need further research into the applicability of these tools in clinical practice across the globe.

## Identification of Risk Factors

As clinicians, we are also interested in risk stratification for a given disorder. There have been various studies that have researched risk factors associated with problematic Internet use and gaming addiction. A Hong Kong study identified social competence and a positive identity to be associated with social networking addiction. Parental roles have also been identified to be crucial in the development of social networking addiction (Yu and Shek). An Indonesian study had also explored the implication of the COVID-19 pandemic on Internet addiction. It found that increased duration of Internet use, internalization, externalization, low prosocial behavior as well as sleep disturbances have been associated with Internet addiction (Siste et al.). A study from Malaysia showed that age, male gender, ethnicity, and psychosocial factors (stress levels, loneliness, depression, anxiety) are also associated with Internet overdependence (Nik Jaafar et al.). Furthermore, a study from China supported the findings of psychological stress being associated with increased problematic smart phone use and social media use (Fung et al.). This information provides opportunities for preventive measures and interventions that target these risk factors.

## Identification of Protective Factors

Studies have also investigated protective factors against the development of Internet and gaming addiction. Two separate studies from Hong Kong had shown that psychological resilience, emotional competence, behavioral competence, beliefs in future as well as spirituality can serve as protective factors (Yu and Shek; Tsui and Cheng). Preventative measures in the form of digital literacy for the public about proper use of the Internet and parental supervision is crucial. Digital competency, which is a step up from literacy, would be pertinent for parents, schools, and healthcare professionals. One needs to be proficient in the use of technology to provide appropriate guidance to the young. It is also important to regulate the use of the Internet in terms of duration and content (10).

## Discussion

A multi-disciplinary approach needs to be adopted in the management of Internet and gaming addiction (Nik Jaafar et al.). Non-pharmacological methods should be prioritized and should include treatment modalities such as psychotherapy, and behavioral interventions (11–13). At this stage, the use of pharmacotherapy is limited as the understanding of the conditions and their underlying mechanisms continue to evolve. There is a need for further research in the field to understand how digital use affects the brains and behaviors of individuals.

## Author Contributions

KV researched on the topic and consolidated it into the first draft. DF provided critical comments and editorial suggestions for revisions. KV then followed up with the changes. All authors agreed on the submitted version.

## Conflict of Interest

The authors declare that the research was conducted in the absence of any commercial or financial relationships that could be construed as a potential conflict of interest.

## Publisher's Note

All claims expressed in this article are solely those of the authors and do not necessarily represent those of their affiliated organizations, or those of the publisher, the editors and the reviewers. Any product that may be evaluated in this article, or claim that may be made by its manufacturer, is not guaranteed or endorsed by the publisher.

## References

[1] Mouratidis K Papagiannakis A. COVID-19, internet, and mobility: the rise of telework, telehealth, e-learning, and e-shopping. Sustain Cities Soc. (2021) 74:103182. 10.1016/j.scs.2021.103182 34540566 PMC8437688

[2] Matamala-Gomez M Bottiroli S Realdon O Riva G Galvagni L Platz T. Telemedicine and virtual reality at time of COVID-19 pandemic: an overview for future perspectives in neurorehabilitation. Front Neurol. (2021) 12:646902. 10.3389/fneur.2021.646902 33841313 PMC8027250

[3] Stiglic N Viner RM. Effects of screentime on the health and well-being of children and adolescents: a systematic review of reviews. BMJ Open. (2019) 9:e023191. 10.1136/bmjopen-2018-023191 30606703 PMC6326346

[4] Lin MP. Prevalence of internet addiction during the COVID-19 outbreak and its risk factors among junior high school students in Taiwan. Int J Environ Res Public Health. (2020) 17:8547. 10.3390/ijerph17228547 33218018 PMC7698622

[5] Kuss DJ Griffiths MD. Social networking sites and addiction: ten lessons learned. Int J Environ Res Public Health. (2017) 14:311. 10.3390/ijerph14030311 28304359 PMC5369147

[6] Shotton MA. Computer Addiction Pb: A Study Of Computer Dependency. 1st ed. Sheffield: CRC Press (1989).

[7] International Statistical Classification of Diseases and Related Health Problems. 11th ed. ICD-11. World Health Organization (2019).

[8] American Psychiatric Association: Diagnostic and Statistical Manual of Mental Disorders: Diagnostic and Statistical Manual of Mental Disorders. 5th ed. Text Revision. Arlington, VA: American Psychiatric Association (2022).

[9] Yoon S Yang Y Ro E Ahn WY Kim J Shin SH. Reliability, and convergent and discriminant validity of gaming disorder scales: a meta-analysis. Front Psychol. (2021) 12:764209. 10.3389/fpsyg.2021.764209 34950088 PMC8689178

[10] Ong SH Tan YR. Internet addiction in young people. Ann Acad Med Singap. (2014) 43:378–82. 25142474

[11] Malinauskas R Malinauskiene V. A meta-analysis of psychological interventions for internet/smartphone addiction among adolescents. J Behav Addict. (2019) 8:613–24. 10.1556/2006.8.2019.72 31891316 PMC7044583

[12] Winkler A Dörsing B Rief W Shen Y Glombiewski JA. Treatment of internet addiction: a meta-analysis. Clin Psychol Rev. (2013) 33:317–29. 10.1016/j.cpr.2012.12.005 23354007

[13] Kim S Noh D. The current status of psychological intervention research for internet addiction and internet gaming disorder. Issues Ment Health Nurs. (2019) 40:335–41. 10.1080/01612840.2018.1534910 30742546

